# Exploring Shamanic Journeying: Repetitive Drumming with Shamanic Instructions Induces Specific Subjective Experiences but No Larger Cortisol Decrease than Instrumental Meditation Music

**DOI:** 10.1371/journal.pone.0102103

**Published:** 2014-07-07

**Authors:** Bruno Gingras, Gerald Pohler, W. Tecumseh Fitch

**Affiliations:** Department of Cognitive Biology, University of Vienna, Vienna, Austria; Max Planck Institute for Human Cognitive and Brain Sciences, Germany

## Abstract

Exposure to repetitive drumming combined with instructions for shamanic journeying has been associated with physiological and therapeutic effects, such as an increase in salivary immunoglobulin A. In order to assess whether the combination of repetitive drumming and shamanic instructions is specifically associated with these effects, we compared the effect of listening to either repetitive drumming or instrumental meditation music for 15 minutes on salivary cortisol concentration and on self-reported physiological and psychological states. For each musical style, two groups of participants were exposed to two conditions: instructions for shamanic journeying or relaxation instructions. A total of 39 participants (24 females) inexperienced in shamanic journeying completed the experiment. Salivary cortisol concentrations were measured before and after exposure to music. In addition, participants filled out a mood questionnaire before and after the experiment and completed a post experiment questionnaire on their experiences. A significant decrease in the concentration in salivary cortisol was observed across all musical styles and instructions, indicating that exposure to 15 minutes of either repetitive drumming or instrumental meditation music, while lying down, was sufficient to induce a decrease in cortisol levels. However, no differences were observed across conditions. Significant differences in reported emotional states and subjective experiences were observed between the groups. Notably, participants exposed to repetitive drumming combined with shamanic instructions reported experiencing heaviness, decreased heart rate, and dreamlike experiences significantly more often than participants exposed to repetitive drumming combined with relaxation instructions. Our findings suggest that the subjective effects specifically attributed to repetitive drumming and shamanic journeying may not be reflected in differential endocrine responses.

## Introduction

Shamanic journeys [1]–[4] are associated with an ancient spiritual practice to reach shamanic trance states, typically described as “journeys to a non-ordinary reality”. The shamanic trance is generally described as an altered state of consciousness (ASC) [5] associated with particular psychophysiological changes such as parasympathetic dominance [6]. According to Tart’s definition [5], altered states of consciousness correspond to “a qualitative alteration in the overall pattern of mental functioning, such that the experiencer feels his consciousness is radically different from the way it functions ordinarily”.

Shamanic practitioners in contemporary Western tradition typically enter trance states through the use of repetitive rhythmic sequences using drums or rattles in a frequency range of 4 to 7 Hz. Music is recognized as a facilitator of ASC [7]. Notably, repetitive drumming has been identified as a form of sonic driving that can facilitate ASC [6], [8]. The frequency range of the rhythmic sequences used to attain ASC has been observed to correspond to that of theta EEG waves [9], and brain wave frequencies have been found to synchronize with rhythmic drumming with repetition rates between 3 and 8 Hz [10]. More recently, Will and Berg [11] reported a significant increase in brain wave synchronization following periodic stimulation with drum sounds and clicks with repetition rates of 1–8 Hz. Michael Harner’s technique of Core Shamanism, probably the best-known shamanistic method in Western practice, is centered on the use of rapid drumming (220 beats per minute, corresponding to a little less than 4 Hz) to attain shamanic trance states [3].

According to shamanic practitioners, the first step in a shamanic journey consists in finding an entrance to the so-called “Lower World”. Once this entrance is found, the participant may encounter his or her “power animal” or other “spirits”, in what is often experienced as a lucid dream. At the end of the journey, participants are led back to “everyday reality” by going back to the “entrance point” and opening their eyes. The instructions associated with the Core Shamanism method, combined with exposure to rhythmic drumming sequences at 8 Hz for 15 minutes, have been shown to be an efficient inducer of ostensibly shamanic imagery [12]–[14]. Shamanic practitioners recommend using live drumming but a CD of monotonous drumming may be used instead [15].

There are few published quantitative studies on the topic of shamanic interventions and shamanic journeys. Earlier EEG studies [9], [10] were criticized because of the uncontrolled movements of the participants [16], [17]. However, Guttmann et al. [18] observed specific brain wave patterns, characterized by low frequency theta waves, in experienced participants using body postures described by Goodman [4], [19] and being exposed to rhythmic rattle sequences. More importantly, shamanistic practices have been reported to induce beneficial therapeutic effects. Harner [20], [21] showed that shamanic journey instructions accompanied with repetitive drumming led to an increase in salivary immunoglobulin A (sIgA) in experienced shamanic practitioners. Pohler et al. [22] showed the benefits of a shamanic intervention done by shamanic practitioners for cancer patients. Shamanic interventions over a period of several months have also been shown to help alleviate pain associated with temporomandibular joint disorders [23].

However, none of these publications reported saliva cortisol measurements. Cortisol is released in response to stress and low levels of blood glucocorticoids [24], [25], and salivary cortisol has been shown to index activation of the hypothalamic-pituitary-adrenal axis [26]. A sudden increase of salivary cortisol is linked with acute stress [27]. Inversely, a decrease in the concentration of salivary cortisol has been associated with relaxation [28], [29], yoga and meditation [30], [31], aromatherapy [32], and, more pertinent to our concern here, exposure to music [33]–[35] and choir singing [36].

In the present study, we sought to investigate more specifically the role of repetitive drumming and of the instructions for shamanic journeying in the induction of both biochemical effects (salivary cortisol concentration), and psychological effects, associated with shamanistic journeying in naïve participants lacking experience in shamanic journeying. To compare the effect of repetitive drumming with other musical styles, participants were exposed either to repetitive drumming sequences or to instrumental meditation music. Because it is a musical style that has been shown to induce a state of relaxation [37] and which, like drumming, does not feature the singing voice, instrumental meditation music provided an appropriate basis for comparison. Additionally, to investigate the role of the shamanistic instructions, participants received either instructions for shamanic journeying (following the Core Shamanism method) or instructions for simply relaxing while listening to the music. This resulted in a two-way factorial design, with four groups of participants: one group received shamanistic instructions and was exposed to repetitive drumming, another one received relaxation instructions and listened to repetitive drumming, a third group received shamanistic instructions and listened to instrumental meditation music, and finally a fourth group received relaxation instructions and listened to instrumental meditation music. Finally, since Hevner [38] and others have shown that the acoustical and structural features of a musical piece tend to be associated with specific emotional states, we briefly considered the possible influence of these features on the physiological and psychological effects induced by our music excerpts. Given that the drumming sequence cannot be characterized in terms of melody, harmony, or mode (major versus minor), we limited our analysis to the spectral (frequency) and rhythmic features of the excerpts.

We hypothesized that participants would report increased feelings of wellbeing and relaxation after music exposure, and that a decrease in salivary cortisol would be observed, in line with earlier reports [33]–[35]. We also surmised that the decrease in salivary cortisol concentration would be more pronounced in the groups receiving instructions for shamanic journeying than in the groups receiving instructions for relaxation. This hypothesis was not based specifically on previous analyses on the effects of shamanic journeying on cortisol levels, due to the dearth of studies investigating these effects, but more generally on studies reporting physiological [20], [21] and therapeutic [23] effects associated with shamanic journeying. Moreover, the subjective experiences reported by participants were expected to be different between the groups receiving instructions for shamanic journeying and the groups receiving instructions for relaxation, based on previous studies reporting the evocation of ostensibly shamanic mental imagery in participants following Harner’s Core Shamanism method [12]–[14].

## Materials and Methods

### Study design

The design included two between-subjects factors, each with two levels: musical style (repetitive drumming versus instrumental meditation music) and type of instructions (instructions to use the music for shamanic journeying versus instructions to use the music for relaxation), yielding four groups of participants. Prior to the main study, a pilot study was conducted to test the experimental procedure with another group of participants.

A power analysis was conducted to estimate the optimal sample size to answer the main hypothesis that cortisol responses would differ between participants exposed to instructions for shamanic journeying and participants exposed to instructions for relaxation, using the software G*Power [39]. This hypothesis corresponds to a within-between interaction in a repeated-measures analysis of variance, with 2 groups and 2 measurements of salivary cortisol concentrations (pre- and post-exposure). For an effect size of 0.20 (partial eta squared or η_ρ_^2^), 40 participants were required to reach 87% power, using an alpha criterion of 0.05 for statistical significance.

### Ethics Statement

The experiment reported in this article was conducted in accordance with the Declaration of Helsinki (revised 1983) and local guidelines of the Faculty of Life Sciences, University of Vienna. According to the Austrian Universities Act 2002 (UG2002), which held at the time the study was carried out, only medical universities were required to appoint ethics committees for clinical tests, application of medical methods, and applied medical research. Therefore, no ethical approval was required for the present study. Written informed consent was given by all participants who could withdraw at any time during the experiment without further consequences. All participant data and personal information were identified by a numeric code and only author G. P. could link the codes to the participants’ names.

### Participants

Participants were recruited either by means of posters or through online advertisements on the homepage of the Department of Cognitive Biology of the University of Vienna. They were invited to participate in a study investigating the effect of different musical styles and experimental instructions on their ability to achieve relaxation. To avoid biasing participants, the text used for recruiting did not mention shamanic journeying. Participants were asked to refrain from taking part in the study if not healthy. Furthermore, participants were instructed not to eat, drink, smoke, or engage in physical activities for at least 30 minutes before the beginning of the experimental session.

Eight participants (5 males, 3 females) were recruited for the pilot study; their age ranged from 23 to 79 years (*M* = 45). Seven of them reported having some experience in shamanic journeying.

Thirty-nine participants, mostly biology students at the University of Vienna, including a few mature students (15 males, 24 females), participated in the main study. None of them were experienced in shamanic journeying. Participants were attributed randomly to one of the four experimental groups. Randomization was done on application time. Group sample sizes varied slightly due to the failure of a few scheduled participants to attend the experimental sessions. The group with shamanic instructions and drum music included 10 participants (3 males, 7 females); their age ranged from 20 to 67 years (*M* = 32). The group with relaxation instructions and drum music comprised 8 participants (4 males, 4 females); their age ranged from 19 to 70 years (*M* = 42). The group with shamanic instructions and instrumental meditation music included 11 participants (3 males, 8 females); their age ranged from 23 to 63 years (*M* = 32). Finally, the group with relaxation instructions and instrumental meditation music comprised 10 participants (5 males, 5 females); their age ranged from 21 to 59 years (*M* = 26). All participants signed a written consent form and received 5 Euros for participating in the study.

### Auditory stimuli and pre-recorded instructions

The repetitive drumming sequence used in this study was excerpted from Michael Harner’s “Shamanic Journey Solo and Double Drumming CD” [40]. We used the second track, which includes a sequence of faster drumming at the end of the 15-minute drumming session, corresponding to the so-called “callback” which acts as a signal to the listener to “come back” from the shamanic journey. This callback signal was replaced by the rattle callback from Michael Harner’s “Shamanic Journey Rattle CD” [41] because the timbral difference makes it is easier to discriminate the callback from the previous repetitive drumming sequence. The total length of the sequence was 15 min 32 sec, including 14 min 58 sec of repetitive drumming.

For the instrumental meditation music excerpt, we selected an excerpt from the third track of the CD “Listening to the Heart” [42]. This track contains a piece of instrumental meditation music, originally composed by Paramahansa Yogananda and recorded in an arrangement for flutes, synthesizer, guitar, bass guitar, and xylophone. The excerpt, which starts at the beginning of the track, is 4 min 58 sec long. We used this specific duration because it corresponded to the end of a musical phrase, accompanied by a reduction in sound intensity, thus providing a smooth continuation. Three successive iterations of the excerpt, each ending with a 4-second fade-out, were repeated to obtain 14 min 55 sec of music. The callback signal was cut and pasted from Michael Harner’s Shamanic Journey Rattle CD, following the procedure described above. The total length of the stimulus was 15 min 28 sec.

All sound stimuli were prepared using Audacity 1.3.12. Stimuli were first equalized using the A-weighted loudness curve, which takes into account the fact that subjective perception of loudness varies according to the frequency range [43] and then equalized for intensity in Praat [44]. A MATLAB script was used to obtain the A-weighted loudness curve.

Experimental instructions (in German) were also pre-recorded using Audacity 1.3.12. This was done to minimize potential nonverbal and verbal effects associated with the experimenter. Both music excerpts and experimental instructions were played from a laptop with external active loudspeakers (M-Audio AV 40) located on a table 1 m above the floor and about 5 m (minimum 3 m) from the participants.

**Table 1:** Mean pre- and post-experiment scores for the subscales of the multidimensional mood questionnaire.

		Positive/negative		Alertness/fatigue		Quietude/disquietude	
Music excerpt	Instructions	Pre	Post	Pre	Post	Pre	Post
Repetitive drumming	Shamanic	30.6 (5.2)	30.6 (6.8)	24.2 (8.3)	24.1 (7.0)	28.3 (7.2)	33.5 (4.9)
	Relaxation	32.5 (6.4)	32.9 (4.3)	21.3 (3.7)	25.4 (3.9)	27.8 (5.2)	33.0 (4.8)
Instrumental meditation	Shamanic	27.3 (6.3)	31.3 (5.5)	27.3 (7.6)	31.0 (4.8)	26.2 (5.3)	30.5 (5.4)
	Relaxation	31.8 (5.8)	31.9 (6.5)	28.2 (7.3)	24.6 (6.2)	32.3 (5.8)	33.4 (5.9)

Standard deviations are indicated in parentheses.

### Data collection

Saliva samples were collected before and after music exposure using the Salivettes (Sarstedt 51.1534.500, Nümbrecht, Germany), a device that consists of a plastic tube containing a cotton wool swab. The concentration of salivary cortisol was analyzed by a bioanalytical procedure. Saliva samples were analyzed using an enzyme immunoassay [45]. This method measures the concentration of a substance in a solution by the use of antigen or antibody reactions [46]. An analysis with a double antibody biotin-linked enzyme immunoassay for cortisol [47], [48] was conducted in the endocrinological lab of the Behavioral Biology Department of the University of Vienna. Inter-assay coefficients of variation were below 10%, and intra-assay coefficients of variation were below 15%.

Mood states were evaluated before and after music exposure using the multidimensional mood questionnaire (Mehrdimensionaler Befindlichkeitsfragebogen), a validated scale in German language that provides subscales for positive/negative mood, alertness/fatigue, and quietude/disquietude [49], [50]. Each subscale is composed of 8 items graded on a 5-point scale. Scores on each subscale are therefore comprised between 8 and 40, with high values corresponding respectively to a positive mood, high alertness, and state of quietude.

An “experience questionnaire” was constructed by author G.P. because a validated scale to assess relaxation experiences and subjective “dreamlike experiences” after the music exposition could not be found in the German language. This questionnaire consisted of five items. The first three items (heaviness, warmth and subjective heart rate) were formulated out of the practice of autogenic training, a relaxation method that aims to influence the autonomic nervous system [51]–[53]. According to Schultz [52], the subjective experience of heaviness and warmth is linked to the change from a normal mental state to an ASC [54]. Heaviness is associated with muscular relaxation [55], whereas warmth corresponds to the relaxation of blood vessels [56], and a decreased heart rate is linked with relaxation and a reduction in anxiety [57]. The fourth item asks for any other body sensations, and the fifth for dreamlike experiences. Additionally, the Neo-FFI [58] was administered to assess the Big Five personality factors, comprising openness, extraversion, conscientiousness, agreeableness, and neuroticism.

### Procedure

To avoid confounding effects due to the circadian and circaseptan rhythms of cortisol secretion, all experimental sessions took place at the same time (19∶00) and on the same day of the week (Wednesday), in an 8.5 m long by 4.6 m wide room (39 m^2^). Participants assigned to the same experimental condition were tested together, in groups of 8 to 11 participants. Participants initially received an information handout instructing them on how to use the Salivettes and describing the body posture to adopt during music exposure. Harner (2006), the founder of Core Shamanism, recommends a specific position for shamanic journeys, which entails lying on the back with the left hand covering the eyes.

Participants were asked to insert the cotton wool swab into their mouth and instructed to chew on the swab for a 3-minute period. Afterwards, the swab was placed back into the tube. Tubes were then immediately placed on ice in a freezer at –20°Celsius. Subsequently, participants were asked to fill out the multidimensional mood questionnaire (form A).

The pre-recorded instructions for either shamanic journeying or relaxation were then played. Briefly, the instructions for shamanic journeying invited participants to lie down using the position specified above, and, using the music as a tool to temporarily alter their state of consciousness, to imagine finding a hole in the ground (such as a crater or a lake), either real or imaginary, from which they would return when hearing the rattle callback. The instructions for relaxation invited the participants to adopt the same position, and then simply to relax while listening to the music until they heard the rattle callback. After listening to the instructions, the participants lay down on blankets and adopted the prescribed body posture.

The 15-minute music excerpt (repetitive drumming or instrumental meditation music) was then played. Afterwards, participants were asked again to provide saliva samples and to fill out the multidimensional mood questionnaire (form B), the experience questionnaire and the NEO-FFI. Participants had the possibility to ask questions or discuss their experiences once the experiment was completed.

### Statistical analysis

All statistical analyses were conducted in SPSS 19 (SPSS Inc., Chicago, IL, USA).

## Results

### Comparison of gender, age, and Neo-FFI factors across experimental groups

Chi-square tests were used to compare the proportions of male and female participants for each experimental condition, and exact tests (two-tailed) were used to compute the significance. No significant differences were found between the groups with instructions for shamanic journeying and the groups with instructions for relaxation, χ^2^(1) = 1.880, *p* = 0.203, between the groups exposed to repetitive drumming and those exposed to instrumental meditation music, χ^2^(1) = 0.003, *p* = 1.000, or among each of the four experimental groups, χ^2^(3) = 1.897, *p* = 0.234.

Because age was not distributed normally among the four experimental groups, a log-transformation was conducted to achieve normality. There was a marginal tendency for age to differ among the four experimental groups, *F*(3,35) = 2.67, *p* = .062. There was also a marginal tendency for participants in the groups exposed to repetitive drumming to be older than those in the groups exposed to instrumental meditation music, *F*(1,35) = 3.88, *p* = .057. Similar results were obtained when conducting the analysis of variance on the untransformed age values (no significant differences were observed with the untransformed values).

Two-way analyses of variance with instructions (shamanic journeying versus relaxation) and music style (repetitive drumming versus instrumental meditation music) as between-subjects factors were conducted to test for differences in NEO-FFI scores and age between experimental groups. The distribution of the NEO-FFI scores did not deviate significantly from normality. None of the scores for each of the five dimensions of the NEO-FFI (neuroticism, openness, agreeableness, extraversion, and consciousness) differed significantly between instructions (all *p*-values >.140) or among the four experimental groups (all *p*-values >.194).

### Analysis of the salivary cortisol concentration

One participant in the group with instructions for shamanic journeying and repetitive drumming exhibited salivary cortisol concentrations that were more than 3 standard deviations higher than the mean and was therefore excluded from further analyses. Because the distribution of the cortisol concentration values deviated significantly from normality, the data was first log-transformed to achieve normality. We then conducted a repeated-measures analysis of variance on the log-transformed values, with music exposure as within-subject factor, and two between-subjects factors corresponding to the instructions and music style.

**Figure 1:**
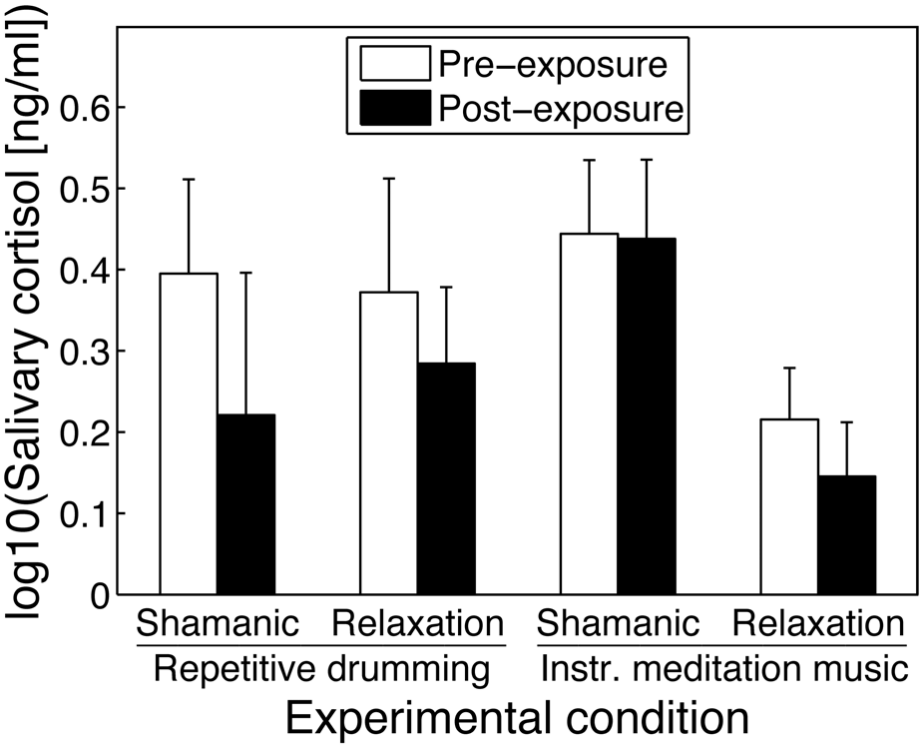
Mean pre- and post-exposure salivary cortisol concentrations for each of the four experimental conditions. *Shamanic*: Instructions for shamanic journeying. *Relaxation*: Instructions for relaxation. *Repetitive drumming*: Repetitive drumming sequence. *Instr. meditation music*: Instrumental meditation music excerpt. Error bars represent the standard error of the mean.

We found a significant main effect of music exposure (Figure 1), representing a decrease in the mean log-transformed salivary concentration of cortisol (in ng/ml) from 0.357 (pre-exposure) to 0.277 (post-exposure), *F*(1,34) = 5.72, *p* = .023, η_ρ_^2^ = 0.14. This corresponds to a reduction of 0.35 ng/ml in the mean untransformed salivary cortisol concentration (from 2.93 to 2.58 ng/ml). No other main effect or interaction reached significance (all other *p*-values >0.179).

### Analysis of the mood questionnaire

To assess the effect of the music on participants’ scores on the multidimensional mood questionnaire, repeated-measures analyses of variance were conducted for each subscale (the data of all groups showed a distribution that did not deviate significantly from normality), with music exposure as within-subject factor (before and after exposure to music for 15 minutes), and instructions and music style as between-subject factors. The scores obtained by each experimental group for the multidimensional mood questionnaire are reported in Table 1.

The mean score on the positive/negative mood subscale increased from 30.4 to 31.6 over all conditions, but this was not statistically significant, *F*(1,35) = 1.545, *p* = .222. No significant main effects or interactions were observed.

A three-way interaction between music exposure, instruction, and music style was found for the alertness/fatigue subscale, *F*(1,35) = 9.40, *p* = .004, η_ρ_^2^ = 0.21. On the one hand, participants who followed the shamanic journey instructions with the instrumental meditation music reported a significant increase in alertness (*p* = .042, Bonferroni-corrected), whereas no significant change in alertness was observed for participants who followed the shamanic journey instructions with the drumming sequence. On the other hand, participants who followed the relaxation instructions with the instrumental meditation music reported a marginally significant decrease in alertness (*p* = .059, Bonferroni-corrected), whereas the opposite was observed for participants who followed the relaxation instructions with the drumming sequence (*p* = .054, Bonferroni-corrected). Additionally, a significant between-subjects effect of music style was observed on this subscale, *F*(1,35) = 4.77, *p* = .036, η_ρ_^2^ = 0.12, mainly because participants (randomly) assigned to the repetitive drumming music groups reported lower pre-exposure levels of alertness, which makes the three-way interaction difficult to interpret.

Finally, we found a significant effect of music exposure on the quietude/disquietude subscale, *F*(1,35) = 14.98, *p*<.001, η_ρ_^2^ = 0.30. Across instructions and music styles, participants averaged higher scores (corresponding to a subjective feeling of quietude) after music exposure (*M* = 32.5) than before music exposure (*M* = 28.6). No other main effect or interaction reached significance.

In summary, the only reliable effect observed on the subscales assessed by the multidimensional mood questionnaire corresponded to an increase in quietude over the course of the experimental session, which did not depend on the instructions received by the participants or on the musical style to which they were exposed.

### Analysis of the experience questionnaire

The experience questionnaire was constructed to assess relaxation experiences and subjective “dreamlike experiences” after the music exposition. The first four items of the scale (heaviness, warmth, heartbeat, and other body sensations) are related to sensations associated with relaxation, whereas the fifth item probes dreamlike experiences (Table 2). Because participants provided binary (yes/no) answers to each item, logistic regression models were applied to each item separately, with instructions and music style considered as predictors.

**Table 2:** Frequency of affirmative responses to the five items of the experience questionnaire.

Music excerpt	Repetitive drumming		Instrumental meditation	
Instructions	Shamanic (10)*	Relaxation (8)	Shamanic (11)	Relaxation (10)
Item				
Heaviness	9 (90.0%)	3 (37.5%)	6 (54.5%)	7 (70.0%)
Warmth	5 (50.0%)	2 (25.0%)	4 (36.4%)	7 (70.0%)
Heart rate	9 (90.0%)	2 (25.0%)	4 (36.4%)	5 (50.0%)
Other sensations	6 (60.0%)	7 (87.5%)	6 (54.5%)	7 (70.0%)
Dreamlike experiences	10 (100%)	2 (25.0%)	8 (72.7%)	9 (90.0%)

*Number of participants for each experimental condition in parentheses.

A significant interaction between music style and instruction was observed for “heaviness”, χ^2^(1) = 5.32, *p* = .021. To investigate this interaction further, we conducted pairwise comparisons between all music style/instruction combinations and applied the Bonferroni correction procedure. A significant effect of instruction was observed for the repetitive drumming sequence: 9 of 10 participants following the shamanic instructions reported experiencing heaviness, versus 3 of 8 participants following the relaxation instructions, *p* = .044 (Bonferroni-corrected). No other significant differences were observed.

Regarding the subjective heart rate question, a significant interaction between music style and instruction was also found, χ^2^(1) = 6.91, *p* = .009. Pairwise comparisons revealed once again a significant effect of instruction for the repetitive drumming sequence: 9 of 10 participants following the shamanic instructions reported a decreased heart rate, versus only 2 of 8 participants following the relaxation instructions, *p* = .003 (Bonferroni-corrected). A significant effect of music style was also observed for the shamanic instruction groups, with only 4 of 11 participants listening to the instrumental meditation excerpt reporting a decreased heart rate, *p* = 0.012 (Bonferroni-corrected).

In the case of the dreamlike experiences, there was also a significant interaction between music style and instruction, χ^2^(1) = 11.63, *p* = .001. Pairwise comparisons showed a significant difference between instructions for the repetitive drumming sequence, *p*<.001 (Bonferroni-corrected), with all 10 participants following the shamanic instructions reporting dreamlike experiences versus only 2 of 8 participants following the relaxation instructions. We also observed an effect of music style for the relaxation instruction groups, *p* = .002 (Bonferroni-corrected), with a higher proportion of participants reporting dreamlike experiences with the instrumental meditation excerpt than with repetitive drumming.

For the “warmth” item, only a marginal interaction between music style and instruction emerged, χ^2^(1) = 3.44, *p* = .063, and no pairwise comparison reached significance. No significant main effect or interaction was found for the “other body sensations” item. To summarize, the largest group differences were observed between the shamanic and relaxation instructions for the repetitive drumming: whereas almost all participants in the group with shamanic instructions and repetitive drumming reported experiencing heaviness, a decreased heart rate, and dreamlike experiences, this was not the case for the majority of the participants in the group with relaxation instructions and repetitive drumming.

### Analysis of the music excerpts

**Figure 2:**
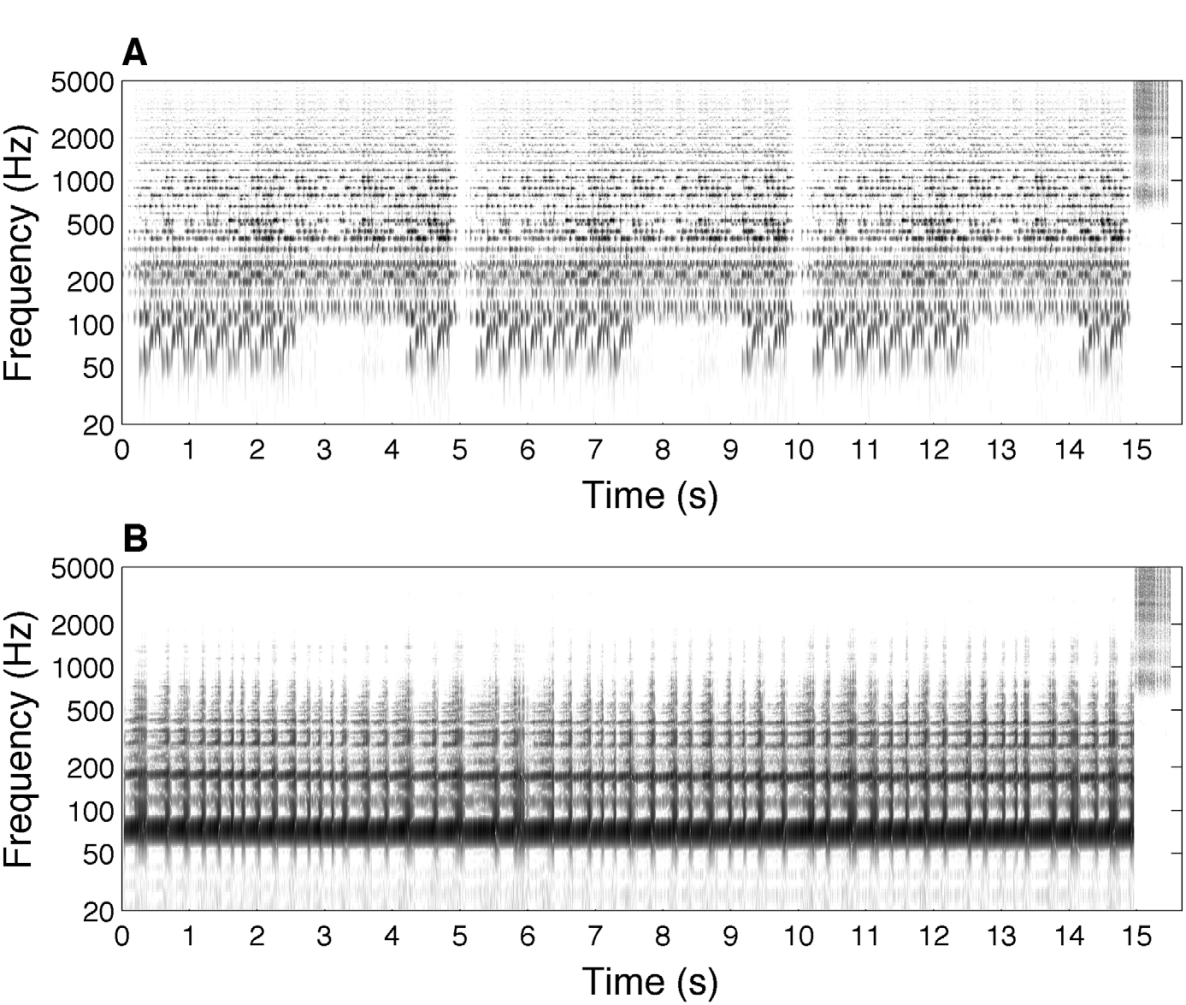
Spectrograms of the music excerpts. A) Instrumental meditation music excerpt. B) Repetitive drumming sequence. Both stimuli end with a 30-second rattle callback. Darker areas correspond to a higher level of energy.

A comparison of the acoustic features of the music excerpts, conducted using the sound analysis software Praat [44], showed that the frequency spectra of the two excerpts were quite different. The energy was spread out over a broad frequency range in the instrumental meditation excerpt (Figure 2A), whereas most of the energy was found below 200 Hz in the repetitive drumming sequence (Figure 2B). The spectrograms also show the more varied content of the instrumental meditation excerpt (as well as the three-fold repetition), compared to the monotonous repetitive drumming. The rattle callback is clearly visible at the end of both stimuli (last 30 seconds). Because the rattle callback is the same for both excerpts, acoustic analyses were conducted separately for the instrumental meditation excerpt (excluding the rattle callback), the repetitive drumming sequence (excluding the rattle callback), and the rattle callback (Table 3). Consistent with the spectrograms (Figure 2), the spectral peak, as well as the center of gravity and standard deviation of the spectrum were much higher for the rattle callback than for the instrumental meditation excerpt and the repetitive drumming sequence, and values were also higher for the instrumental meditation excerpt than for the repetitive drumming sequence.

**Table 3:** Spectral features and rhythmic characteristics of the music excerpts.

Music excerpt	Instrumental meditation	Repetitive drumming	Rattle callback
Center of gravity of spectrum (Hz)	744.4 (469.6)	126.8 (115.6)	5505.2 (2859.7)
Spectral peak (Hz)	392.1	71.2	2751.9
Beat duration (s)	1.228 (0.018)	0.236 (0.001)	0.191 (0.026)
Tempo (beats per minute)	48.9 (0.7)	254.7 (1.3)	314.4 (4.3)
Inter-onset interval (s)	0.313 (0.215)	0.236 (0.01)	0.095 (0.026)

Standard deviations are indicated in parentheses. For the rattle callback, the tempo was calculated only for the middle section given that the beginning and end of the callback were irregular.

The rhythmic characteristics of the excerpts, specifically the mean tempo and inter-onset interval (i.e., the time interval between successive note onsets), were determined using the Tempogram Toolbox in MATLAB [59] and verified by visual inspection of the amplitude envelopes (Table 3). The mean tempo was much faster (254.7 beats per minute [BPM], corresponding to 4.2 Hz) for the repetitive drumming than for the instrumental meditation excerpt (48.9 BPM, or 0.8 Hz). The coefficient of variation of the tempo (standard deviation divided by the mean tempo, expressed in percentage), which provides an indication of the stability of the tempo, was slightly higher for the instrumental meditation excerpt (1.4%) and for the rattle callback (1.4%) than for the repetitive drumming sequence (0.5%). In addition, the instrumental meditation excerpt exhibited a much greater rhythmic variety than the drumming sequence, as indicated by a comparison of the standard deviation of the inter-onset intervals.

## Discussion

In this study, we evaluated whether listening to 15 minutes of repetitive drumming or instrumental meditation music, while lying down, with instructions for shamanic journeying led to different effects on salivary cortisol and emotional states compared with listening to the same music excerpts with instructions for relaxation only. The main hypotheses were that after exposure participants would report increased feelings of wellbeing and relaxation, and that a decrease in salivary cortisol concentration would be observed. The reduction in salivary cortisol concentration was predicted to be stronger for the experimental groups with instructions for shamanic journeying. Moreover, reported experiences were expected to be different for the groups with instructions for shamanic journeying compared to the groups with instructions for relaxation.

We found a significant effect of the overall treatment on salivary cortisol concentration, with lower cortisol concentrations post-exposure, but no significant interactions with instructions or music style. The observed decrease in cortisol concentrations post music exposure is in line with previous observations [35]. Moreover, the magnitude of the decrease (−0.35 ng/ml) was comparable to that observed in participants after one hour of music listening (−0.33 ng/ml) [36] or after a 15-minute conversation with a remote partner using a huggable human-shaped device (−0.50 ng/ml) [60]. Here, we found that only 15 minutes of exposure to either repetitive drumming or instrumental meditation music, while participants lay on their backs, are sufficient to induce a significant decrease in salivary cortisol concentration, thus corroborating our first hypothesis. However, no additional effect on salivary cortisol concentration associated specifically with exposure to repetitive drumming or to shamanic journeying instructions was detected, thus disconfirming our second hypothesis.

The analysis of the mood questionnaire subscales revealed a significant increase on the quietude/disquietude subscale, suggesting that, as predicted by our first hypothesis, participants felt calmer and more relaxed after music exposure, regardless of the instructions or music style. This is in line with previous studies in music research [33]–[35]. There was no significant effect associated with the positive/negative mood subscale. We observed a complex three-way interaction on the alertness/fatigue scale, but this effect is difficult to interpret and probably irrelevant due to pre-experimental differences in the level of alertness between the participant groups (even though participants were randomly assigned to the groups).

The analysis of the experience questionnaire revealed a significant interaction between experimental instructions and musical style for three items. Most of the participants exposed to repetitive drumming and shamanic instructions reported experiencing heaviness, a decreased heart rate, and dreamlike experiences, whereas the majority of the participants exposed to repetitive drumming and relaxation instructions did not report these experiences. These findings suggest that shamanic instructions combined with repetitive drumming may lead to a greater likelihood of subjective experiences associated with relaxation, decreased arousal levels, and dreamlike states, when compared to other musical styles or instructions.

Because the shamanic instructions given to the participants may have suggested specific subjective experiences [8], the fact that these experiences differed between participants exposed to shamanic instructions and those exposed to instructions for relaxation is not entirely unexpected. However, these different outcomes were only observed for the groups exposed to repetitive drumming, whereas no significant differences emerged between these two instructions for the groups exposed to instrumental meditation music. Although very few studies have investigated this phenomenon systematically, participants exposed to repetitive drumming while completing an imagery task reported significantly different subjective experiences in comparison to participants who completed the imagery task without exposure to drumming [61]. Furthermore, differences between subjective experiences related to shamanic instructions and other instructions, such as sitting quietly, were previously reported by Rock [12], [13]. In a similar vein, Shapiro and Lehrer [62] found that participants given autogenic training (which has some similarities with shamanic journeying) reported more sensations of warmth and heaviness in the limbs than participants in a progressive relaxation group. However, no differences in heart rate were found between both groups.

Given that the music excerpts used in our study differed substantially in terms of their spectral and rhythmic features, we might have expected to find a main effect of musical style, especially with respect to the mood and experience questionnaires. Indeed, music characterized by a high frequency range and a varied rhythm is generally perceived as happy and graceful, whereas a low frequency range and an unvaried rhythm tends to be perceived as solemn and heavy (see e.g., [38]). Here, the frequency range for the instrumental meditation excerpt was substantially higher than that of the repetitive drumming sequence. Moreover, although the former exhibited a slower tempo, its rhythmic variety was much greater than that of the strictly isochronous drumming sequence. Nevertheless, we did not observe any significant main effect of musical style, either on the salivary cortisol concentration or on the responses to the questionnaires. To be sure, these findings are far from conclusive, considering that we only compared drumming to meditation music, and a broader repertoire should be explored to fully investigate the impact of various musical styles.

In summary, our results indicate that, whereas differences in subjective experiences were observed between experimental conditions, no significant differences were observed in the salivary cortisol response, suggesting that the subjective experiences specifically attributed to repetitive drumming and shamanic journeying may not be reflected in differential endocrine responses. Although there are few comparable studies in the literature, Nater et al. [63] also reported no significant treatment × condition interaction for salivary cortisol levels between two groups of participants exposed to heavy metal (pre-selected as arousing and unpleasant) versus Renaissance music (pre-selected as relaxing and pleasant), in spite of differential effects for psychological responses such as the positive/negative mood and quietude/disquietude subscales (this study used the same multidimensional mood questionnaire employed here). However, the same study also reported significant differences for other psychophysiological responses such as heart rate and skin conductance, indicating that differential effects observed on psychophysiological measurements may not be reflected in endocrine variables.

In contrast to related studies (e.g., Thoma et al. [64]), we did not attempt to control for all potential confounding variables. Hence, our sample of participants exhibited a large range of variation in age, and included participants from both genders. The latter is a potentially confounding factor given that it has been shown that males and females differ in their physiological and emotional responses to music listening [63], [65]. While this may have reduced the internal validity of our study, it also suggests that our conclusions may be generalized to a broader population. Furthermore, we note that no statistically significant differences were observed between experimental conditions for either age (although a marginal tendency was found in this case) or for the proportion of male/female participants. To be sure, we cannot exclude the possibility that using a different experimental design might have yielded different results. For instance, participants were tested in small groups in our study, as in Knight & Rickard [66], although a meta-analysis has suggested that music interventions might be more effective with individuals than with groups [67].

As shown in earlier studies [14], [68], [69], the composition of the participant groups and the setting (environment and circumstances) can have a strong influence on the outcome of shamanic journeying. The intentions and goals associated with the journey, as well as the personality traits, psychological states, or belief systems of the participants may play an important role in the personal experiences and physiological changes associated with exposure to repetitive drumming and/or instructions for shamanic journeying. Besides the physical setting, the presence of live drumming or rattling versus the use of recorded music, or the presence of one or more experienced shamanic practitioners may potentially lead to different outcomes. Future studies may compare the effects experienced by shamanic practitioners to those experienced by naïve participants. Furthermore, although the reported association between repetitive drumming and ASCs was the motivation for our use of repetitive drumming, other musical styles with contrasting rhythmic or timbral features should be employed in order to assess the impact of different acoustic parameters in a more systematic fashion. Finally, other physiological markers besides cortisol, such as IgA, salivary alpha-amylase, testosterone, or possibly oxytocin, could be analyzed from saliva samples.

## Supporting Information

Table S1 **Experimental data collected for each individual participant in the main study.** The data includes the following: salivary cortisol concentration, inter-assay coefficient of variation for the salivary cortisol measurements, responses to the multidimensional mood questionnaire, responses to the experience questionnaire, and NEO-FFI scores. Each row represents the data for an individual participant. The participants’ age is not included in order to preserve their anonymity.
(XLSX)
